# qtl2pleio: Testing pleiotropy vs. separate QTL in multiparental populations

**DOI:** 10.21105/joss.01435

**Published:** 2019-06-30

**Authors:** Frederick Boehm, Brian Yandell, Karl W. Broman

**Affiliations:** 1 Department of Statistics, University of Wisconsin-Madison; 2 Department of Biostatistics and Medical Informatics, University of Wisconsin-Madison

## Abstract

Modern quantitative trait locus (QTL) studies in multiparental populations offer opportunities to identify causal genes for thousands of clinical and molecular traits. Traditional analyses examine each trait by itself. However, to fully leverage this vast number of measured traits, the systems genetics community needs statistical tools to analyze multiple traits simultaneously (Jiang & Zeng, 1995; Korol, Ronin, & Kirzhner, 1995). A test of pleiotropy vs. separate QTL is one such tool that will aid dissection of complex trait genetics and enhance understanding of genetic architecture.

Jiang & Zeng (1995) developed a pleiotropy test for two-parent crosses. For a pair of traits that map to a single genomic region, they formulated the test with the null hypothesis being pleiotropy (the two traits are affected by a single QTL) against the alternative hypothesis of two separate QTL, with each QTL affecting exactly one trait in the pair.

The test of Jiang & Zeng (1995) doesn’t directly apply to multiparental populations because

1. Multiparental populations have more than two founders
2. Multiparental populations have complicated pedigrees

Additionally, the test statistic distribution, under the null hypothesis of pleiotropy, doesn’t follow a distribution with tabulated quantiles, like the chi-square with 1 degree of freedom. Thus, we need to implement a method for determining p-values.

We addressed the first two challenges by adding a fixed effect for every founder line and incorporating a multivariate polygenic random effect into the linear model, which resulted in a multivariate linear mixed effects model (Kang et al., 2008; Zhou & Stephens, 2014). We implemented a parametric bootstrap procedure to determine p-values for test statistics (Efron, 1979; Tian et al., 2016). We describe details of our statistical methods elsewhere (Boehm, Chesler, Yandell, & Broman, 2019).

qtl2pleio offers a convenient interface for those already analyzing data with qtl2. The primary functions in qtl2pleio are scan_pvl, to perform the multivariate multi-QTL scan, and boot_pvl, to obtain bootstrap samples. We also include functions for visualizing results. qtl2pleio features three R package vignettes that demonstrate these and other qtl2pleio functions. One vignette provides examples for performing bootstrap analysis with a computing cluster. For quality assurance purposes, we incorporated unit tests into qtl2pleio via the R package testthat (Wickham, 2011).

qtl2pleio uses C++ code for model fitting via generalized least squares. We use the R package Rcpp to interface with our C++ code (Eddelbuettel et al., 2011). We also make use of the C++ library Eigen via the R package RcppEigen (D. Bates & Eddelbuettel, 2013).
